# Final Oocyte Maturation in Assisted Reproduction with Human Chorionic Gonadotropin and Gonadotropin-releasing Hormone agonist (Dual Trigger)

**DOI:** 10.5935/1518-0557.20160047

**Published:** 2016

**Authors:** Sofia Andrade de Oliveira, Vinícius Fernando Calsavara, Gemma Castillón Cortés

**Affiliations:** 1 Cenafert - Reproductive Medicine Center, Salvador, BA, Brazil; 2 São Paulo University - USP, São Paulo, SP, Brazil; 3 Valencia Infertility Institute - IVI, 14 Ronda del General Mitre, Barcelona, Spain

**Keywords:** Assisted Reproduction, GnRH Agonist Trigger, hCG Trigger, Poor Responder, Immature Oocyte, Ovarian Hyperstimulation Syndrome

## Abstract

Final oocyte maturation with Human Chorionic Gonadotropin (hCG) and ovarian stimulation with Follicle Stimulation Hormone (FSH) combined with Gonadotrophin-releasing Hormone (GnRH) antagonist to block Luteinizing hormone (LH) surge is a standard procedure of in vitro Fertilization (IVF) and Intracytoplasmic Sperm Injection (ICSI). However, GnRH agonist has been replacing the use of hCG in certain situations, especially in patients at risk of Ovarian Hyperstimulation Syndrome (OHSS). Some studies have also shown advantages in the combined use of GnRH agonist concurrently with hCG in inducing final oocyte maturation, a treatment known as "Dual Trigger". In theory, this method combines the advantages of both induction regimens, and it has brought promising results. The objective of this study is to compare Dual Trigger with the use of hCG alone or the use of GnRH agonist alone. A systematic review of articles on Dual Trigger and a retrospective cohort study comparing the three methods of induction of final oocyte maturation have been conducted. It has been found that Dual Triggering for poor responder patients had a statistically significant increase in the number of retrieved oocytes, mature oocytes, and fertilized embryos in the positive beta hCG rate, implantation rate, and newborn/transferred embryo (TE) rate.

## INTRODUCTION

Final oocyte maturation with hCG and ovarian stimulation with FSH combined with GnRH antagonist to block LH surge is a standard procedure of in vitro Fertilization (IVF) and Intracytoplasmic Sperm Injection (ICSI) (Decleer *et al*., 2014). Human Chorionic Gonadotropin is routinely used for inducing LH surge, thus inducing final oocyte maturation (Schachter *et al*., 2008). However, the use of hCG can result in Ovarian Hyperstimulation Syndrome (OHSS) (Shapiro *et al*., 2008). This risk is significantly reduced by replacing hCG with a GnRH agonist (Shapiro *et al*., 2008; Zilberberg et al., 2015; Lin *et al*., 2013). The short half-life of pituitary LH combined with the desensitization induced by the agonist results in a rapid and irreversible luteolysis, ideally eliminating the risk of OHSS (Shapiro *et al*., 2011; Griffin *et al*., 2012). In addition to that, some studies have shown that administering GnRH agonist after the use of GnRH antagonist in an IVF cycle brings about true benefits for implantation, since the antagonist blocks endometrial GnRH receptors, worsening endometrial quality. Once the GnRH agonist - that has a much higher affinity to receptor than the GnRH antagonist - is administered, a displacement of the antagonist from the receptor occurs in the endometrium, and it unlocks these receptors, improving endometrial receptivity (Schachter *et al*., 2008).

However, as stated earlier, the rapid luteolysis caused by the use of the agonist consequently leads to an altered luteal phase, and its final result is the reduction of implantation rates and the increasing of miscarriage rates, when compared to the use of hCG as a "trigger" (Lin *et al*., 2013; Griffin *et al*., 2012). To solve this problem, some studies have shown that it was possible to improve implantation rates by administering high doses of progesterone alone - or combined with estrogen - on the luteal phase after using the GnRH agonist (Shapiro *et al.*, 2011; Griffin *et al.*, 2012). Another possibility would be to transfer the vitrification of embryos into another cycle with more appropriate hormone levels. Furthermore, in an effort to reduce miscarriage rates, few studies have evaluated the effects of the use of GnRH agonists associated with hCG 12-35 hours after triggering with the agonist, and they have shown some improvements in the luteal phase (Humaidan, 2009). However, the subsequent hCG administration does not act as oocyte maturation (Shapiro *et al*., 2008).

For approximately eight years, some studies have shown a fourth possibility: the combination of the use of GnRH agonist concurrently with hCG to induce final oocyte maturation (Griffin *et al*., 2012), a treatment known as "Dual Trigger", which has been used in patients with high response, normal response, and poor response, or oocyte immaturity.

In theory, this method combines the advantages of both induction regimens:

1. it decreases the risk of OHSS by decreasing the dose of hCG;
2. it tends to be a more physiological cycle since there is an FSH peak addition induced by GnRH agonist generating a larger number of mature oocytes (Griffin *et al*., 2014; Haas *et al*., 2014; Castillo *et al*., 2013), whereas hCG alone induces LH peak (Decleer *et al.*, 2014);
3. it improves endometrial receptivity for releasing endometrial GnRH receptors;
4. it extends the ovulation time after use of the inductor caused by hCG, also improving the maturation (Zilberberg *et al*., 2015);
5. there is a better luteal phase recruitment when there is a proven combined use of hCG with GnRH agonist (Shapiro *et al*., 2011).

However, in practice, there is a statistically significant difference between the induction of oocyte maturation with isolated hCG, or isolated GnRH agonist, and the induction of GnRH agonist combined with hCG in terms of oocyte numbers, embryo quality, and clinical results?

The objectives of this study are:

1. to evaluate "Dual Trigger" studies in high responder patients, poor responders or patients with oocyte immaturity, and normal responder patients;
2. to retrospectively evaluate the results obtained with this treatment in the Valencian Infertility Institute, in Barcelona, Spain.

## MATERIAL AND METHODS

### Systematic Review

During June 2016, we carried out a systematic review in Pubmed database using as descriptors (Mesh) the words: "Gonadotropin-Releasing Hormone / agonists" AND "Chorionic Gonadotropin, Human". In total, we found 319 papers. Notwithstanding, we had to discard 306 papers for the following reasons: 28 were experiments on animals; nearly 170 papers discussed the use of hCG alone as a "trigger"; 96 argued about the use of GnRH agonist alone as a "trigger", and 12 addressed the use of hCG during the luteal phase, or after the use of GnRH agonist. We ended up with thirteen papers on the use of Dual Trigger: 6 prospective studies (3 randomized), 6 retrospective cohort studies, and one case report. To report the results of this systematic review we used the Preferred Reporting Items for Systematic Reviews and Meta-Analysis (PRISMA) statement, by Moher *et al*., 2010.

### Retrospective Cohort Study

In addition to the systematic review, a retrospective cohort study was carried out with data collected from electronic medical records of a Human Reproduction Clinic in Barcelona, Spain, from June 2014 to March 2015, with high and poor responder patients who had been treated with Dual Trigger. Dual Triggering has been compared to conventional treatment (hCG alone, in normal or poor responder patients, and the use of GnRH agonist in high responders) after controlling ovarian stimulation with FSH + LH, with doses set based on patient's weight, age and history, and the use of GnRH antagonist to block the premature LH surge. All patients had the luteal phase support with progesterone (Utrogestan^®^ 1200mg/day, beginning on the day after the ovarian puncture), and all patients were 40 years old or less, according to the inclusion criteria. High responder patients were classified according to the following criteria: more than 20 follicles > 12 mm during controlled ovarian stimulation, or estradiol > 3000pcg/ml, or risk factors for OHSS. Among all patients, those who had been treated with concomitant induction of final oocyte maturation with hCG + GnRH agonist (Group A: GonasiR 2,500 IU + DecapeptylR 0.2mg) - that is, Dual Trigger - were compared to the control group, patients who received only GnRH agonist (Group B: DecapeptylR 0.2mg). Poor responder patients who classified according to the Bologna criteria were also compared to patients who had been administered hCG + GnRH agonist (Group C: OvitrelleR 6,500 IU + DecapeptylR 0.2mg) concurrently with the control group of patients who had received hCG alone (Group D: OvitrelleR 6,500 IU) as a trigger.

### Statistical analysis

Initially, we carried out a descriptive analysis of the data from which the quantitative variables were evaluated. In order to assess whether there was a correlation between quantitative variables, we used the Pearson correlation coefficient. Some parametric techniques were used in cases where data normality had been met; however, nonparametric tests were applied when normality assumption had been violated. In order to test hypotheses by comparing the averages of two independent samples, the t-test or the Mann-Whitney U test was applied. For data where repeated measures were found for the same variable, we used the nonparametric ANOVA test. For all tests, we considered a significance level of 5%, and a confidence interval of 95%. The analyses were performed by means of the R Core Team (2014) and the Statistical Package for Social Sciences (SPSS 18, SPSS Inc., Chicago, IL, USA).

### Ethics Procedures

Ethics Approval was required for it is a retrospective study and a systematic review using the electronic medical records of the Valencian Infertility Institute, where our research was carried out. The patients did not have their names disclosed or their behaviors modified. The type of treatment investigated in this study has already been discussed in other studies, including prospective and randomized investigations that have shown positive outcomes.

## RESULTS

### Systematic Review

Five articles comparing the use of GnRH agonist alone with Dual Trigger in high responder patients were found: four retrospective cohort study papers and one prospective study (see Table 1).

**Table 1:** Articles on High Responders Patients

Article	N	Patients	Groups	Luteal Support	Statistically significant results (p<0.05)
Shapiro B. et al. 2011. **USA** **Retrospective** **cohort study**	273	High Responders	G1) Dual Trigger, G2) Agonist only; G3) Agonist only + intensive luteal support Agonist: Leuprolide acetate. 4mg hCG: 20UI/Kg ( 1000- 2500UI)	E_2_, 0,2mg/72h + E_2_ 6mg/d + P4, 100mg /d, IM + P4, 800mg/d, VV, beginning 2-5 days after retrieval. Intensive luteal support: beginning immediately after retrieval.	G1: < number of Retrieved Oocytes (20.4±6.2 vs. 27.1±11.2 vs. 25.4±10.3); > Implantation rate (%) (48.8 vs. 20.6 vs. 37.8); > Pregnancy rate (%) (75.3 vs. 60.4 vs. 15); > ongoing pregnancy rate (%) (57.7 vs. 25.3 vs. 50).
O'Neill K.E. et al. 2016, **USA** **Retrospective** **cohort study**	174	High Responders	G1) GnRH agonist: G2) GnRH agonist + hCG Agonist: leuprolide acetate. 4mg hCG: 1000UI	It started on the day after oocyte retrieval: daily IM, 50 mg progesterone 50 mg, IM/day + three 0. 1mg transdermal patches of estradiol, daily	G2: > % oocyte maturity (70% vs. 82%. <% oocytes retrieved (0% vs 3%), > % OHSS (6% vs 0%) and severe OHSS (4% vs. %)
Jung Y.H. et al. 2014. **Korea** **Prospective** **cohort study**	26	High responders	G1) GnRH agonist: G2) GnRH agonist + hCG 1000IU; G3) GnRH agonist + hCG 20001U.	Progesterone (dose: is not described)	G1: < number of retrieved oocytes (4.7±1.5 vs. 10.0±3.7 vs. 20.2±6.8); < pregnancy rate (%) (14,3 vs. 75 vs. 70)
Griffin D. et al. 2012. **USA** **Rctrospcctivc** **cohort study**	102	High Responders	G1) Dual Trigger; G2) GnRHa alone. Agonist: Leuprolide acetate. 1mg hCG: 1000IU	P4, 50mg/d, IM + E_2_, transdermal, 0,3mg/2d	G1 : < number of embryo frozen (4.3±4.7 vs. 3.6±3.1 ): < Implantation rate (%) (22.1 vs. 41.9); < Pregnancy rate (%) (36.8 vs. 58.8): < live birth rate (%) (30.9 vs. 52.9)
Sherhahn R. 2015. **USA** **Retrospective** **cohort study**	135	Highre sponders	G1)GnRG Agonist; G2) Dual Trigger. hCG: 1500-2000IU. Agonist: Lupron.	P4. IM + E_2_ Oral or transdcrmal	No statistically significant results.

The results of these studies were well mixed. While the study by Shapiro *et al*. (2011) showed higher implantation and pregnancy rates in the Dual Trigger group and a lower number of retrieved oocytes and embryo transfers in this same group, the study by Griffin *et al.* (2012) showed lower implantation and pregnancy rates in patients who were treated with the Dual Trigger modality. The study by Sherbahn & Catenacci (2015) showed no statistically significant difference between the use of Dual Trigger and isolated GnRH agonist. Jung *et al*. (2014) analyzed a small sample size of 26 patients only and found that dual Triggering with a 2000IU dose of hCG produced a higher pregnancy rate. The study by O'Neill *et al*. (2016) showed a higher risk of OHSS in high responder patients who had been treated with the Dual Trigger mode.

Regarding the group of normal responder patients (Table 2), we found 4 papers comparing the use of hCG alone with the Dual Trigger mode: 3 prospective and randomized, and one retrospective cohort study. In this group, the results were quite uniform, and they showed that, by using the Dual Trigger strategy, there was a higher number of collected oocytes and mature oocytes, as well as good quality embryos. They have also shown an increase in implantation and pregnancy rates.

**Table 2:** Articles on Normal Responder Patients

Article	N	Patients	Groups	Luteal Support	Statistically significant results (p<0,05)
Decleer W. et al. 2014, **Belgium** **Prospective** **randomized**	120	Normal Responders	G1) hCG alone; G2) Dual Trigger hCG: Pregnyl^R^ 5000UI Agonist: Gonapeptyl^R^ 0.2mg	Utrogestan^R^, 600mg. vv	G2: > number of patients with at least one top quality embryo (%) (73.8 vs. 47.5); > number of patients with embryos for cryopreservation (54.1 vs. 35.6)
Kim C.H. et al. 2014. **Korea** **Prospective** **Randomized**	120	Normal Responders	G1) Dual Trigger G2) hCG alone. (Doses: not related)	Progesterone (Doses: is not described)	G1 : >Implantation rate (%) (24.7 vs. 14.9); > Pregnancy rate/cycle (%) (53.3 vs. 33.3); > live birth rate (%) (50.0 vs. 30.0)
Lin M.H. et al. 2013, **Taiwan** **Retrospective** **Cohort**	376	Normal Responders	G1) Dual Trigger; G2) hCG alone hCG: Ovidrel^R^, 250ug Agonist Decapeptyl^R^ 0,2mg	P,50mg,d, IM + Utrogestan^R^, 300mg, vv	G1: > number of retrieved oocytcs (12.36±6.64 vs 10.10±4.58); > number of oocytes MII (10.531±6.47 vs. 8.03±4.51);> number of cryopreserved embryo (1.97±0.12 vs. 1.601±0.49); > Implantation rate (%) (29.68 vs. 18.43); > Pregnancy rate/Embryo Transfer (%) (41.36 vs. 30.49)
**Bulut H. et al. 2015**. **Turkey** **Prospective** **Randomized.** **Double Bind**	400	Normal Responders	G1) Dual Trigger; G2) hCG alone. hCG: Ovitrclle^R^ 6.500IU; Agonist: Triptorelin acetate. 0,2mg	Progesterone (Dose: ?)	It has not been published yet.

Finally, 3 papers comparing the use of hCG alone with Dual Trigger in poor responder patients or with immature oocyte and found: 2 prospective, and one retrospective study. In this case, the results were uniform as well, showing improvements in the number of oocytes, mature oocytes, fertilized embryos, and good quality embryos, as well as an increase in the implantation and pregnancy rates using Dual Trigger (Table 3).

**Table 3:** Articles on Poor Responders Patients or Patients with Imature Oocytes.

Article	N	Patients	Groups	Luteat Support	Only Statistically Signiticant Resutts (p<0,05)
Schachter M. et al. 2008, **Israel,** **Prospective** **randomized**	200	Poor Responders	G1): Dual Trigger: G2): hCG only. hCG: Chongon^R^. 5000UI Agonist: Diphereline^R^, 0,2mg	Utrogestan^R^, 400mg. vv	G1: > Pregnancy rate/Embryo transfer (%) (44.3 vs. 29.1); > Ongoing pregnancy rate per Embryo transfer (%) (36.1 vs. 22.3)
Griffin D. et al, 2014, **USA** **Retrospectiv** **e Cohort**	54	>25% immature oocytcs	G1) Dual Trigger hCG 5.000 IU + Leuprolide acetate. lmg:G2) hCG 10.000UI only.	Progesterone. 50mg/d, IM	G1: > number of mature oocytes retrieved (%) (75 vs. 38.5)
Zilberberg E. et al. 2015 , **Israel** **Prospective** **Study**	24	>34% immature oocytcs	G1) Dual Trigger: G2) hCG only. (Doses: is not described)	Progesterone	G1: > number of mature oocytes (n) (7±3.3 vs. 3.6±2.2): > number of embryos transferred (n) (2.2±1.0 vs. 1.1±1.0); > number of top quality embryos (n) (3.1±2.7 vs. 0.8+1.5)

### Retrospective cohort study

As described in the methodology section, we designed a retrospective cohort study, comparing two groups of high responder patients (Dual Trigger x GnRH agonist alone) and two groups of poor responder patients (Dual Trigger x hCG alone).

### A) High Responder patients

The group of high responders consisted of a total of 24 patients, 12 in group A (Dual Trigger) and 12 in B (GnRH alone). There was no statistically significant difference in terms of age, BMI, basal FSH, AMH, antral follicle count, stimulation duration, level of estradiol, and endometrial thickness on the trigger of patients from both groups.

There was no statistically significant difference among collected oocyte numbers, number of mature oocytes, fertilized embryos, good quality embryos, transferred embryos, or vitrified embryos.

Group A (Dual Trigger) showed higher implantation and newborn/transferred embryo rates with statistical significance (table 4).

**Table 4:** High Responders.

Variable	Dual Trigger (N = 12)	GnRlla alone (N = 12)	*P*
Bela hCG + Rate	(6/12) 50,00%	(6/12) 50,00%	NS
Implantation Rate	(9/13)69,23%	(8/18) 44,44%	<0,05
Newbom/ET	(9/13) 69,23%	(7/18) 38,89%	<0,05

### B) Poor Responder Patients

Poor responders were composed of a total of 40 patients divided in two groups as follows: 18 patients in group C (Dual Trigger) and 22 patients in group D (control group). Comparing both groups, there were no statistically significant differences in terms of age, BMI, basal FSH, AMH, antral follicle count, stimulation period, level of estradiol, and endometrial thickness on the day of trigger between patients from groups C and D.

Corroborating the results of other studies in the systematic review, this retrospective study has shown a higher number of oocytes retrieved, mature oocytes, and embryos fertilized, with statistically significant difference in patients who were treated with Dual Trigger (Table 5).

**Table 5:** Poor responder Patients.

Variable	Dual Trigger(N- 18)	hCG alone(N* 22)	*P*
Oocytcs	7,03 ± 3,12	4,67 ± 1,63	<0.05
Oocytcs MII	5,38 ± 2,82	3,32 ± 1,24	<0.05
Fertilized Embryos (2 PN)	4,02 ± 2,34	2,80 ± 1,43	<0.05
Top Embryos	2,12 ± 1,68	1,93 ± 1,45	NS
Vitrificated Embryos	0,83 ± 1,36	0,50 ±0,44	NS
Transferred Embryos	1,45 ± 0,82	1,53 ± 0,93	NS

**Variable**	**Dual Trigger** **(N= 18)**	**hCG alone** **(N=22)**	***P***
Beta hCG + Rate	(9/18) 50,00%	(6/22) 27,27%	NS
Implantation Rate	(12/27)44,4%	(6/31) 19,35%	<0,05
Newborn/ET	(12/27)44,4%	(5/31) 16,13%	<0,05

There was no difference between the numbers of transferred or vitrified evolutionary embryos, but group C (Dual Trigger) produced higher implantation and newborn/ transferred embryo rates.

## DISCUSSION

This article shows that the use of GnRH agonist combined with hCG in inducing final oocyte maturation is an excellent alternative after ovarian stimulation with recombinant FSH and LH, and suppression of premature LH surge with GnRH antagonist, especially in normal responder patients, poor responder patients, or patients with immature oocytes. Our retrospective study and systematic review demonstrated that there is yet no indication of the use of Dual Triggering mode in high responder patients, since the results of these studies were contradictory, and it was impossible to assess the risk of OHSS due to the low prevalence of this disorder. Supporting the theory explained in the introduction of this paper, in the cases of normal and poor responder patients, all reviewed studies - including this retrospective study - have shown the superiority of treatment with Dual Trigger regarding the number of mature oocytes, fertilized embryos, and regarding the implantation rate and the newborn/transferred embryo rate (Decleer *et al*., 2014; Schachter *et al*., 2008; Shapiro *et al*., 2008; Zilberberg *et al*., 2015; Lin *et al*., 2013).

The Dual Trigger strategy significantly improved embryo quality, it would be interesting to carry out the work in patients with low embryo quality to assess their actual effectiveness. Furthermore, this method probably has an advantage in cases of egg donation, to increase the number of oocytes in normal responder patients. However, we must be careful, for it is not possible to assess whether Dual Trigger increases the risk of OHSS when compared to the GnRH agonist alone (Engmann *et al*., 2008). Moreover, O'Neill *et al.* (2016) showed that the risk of OHSS has been increasing in high responder patients treated under the Dual Trigger strategy.

It is necessary to compare the use of Dual Trigger with hCG alone in vitrified embryo cycles followed by vitrified embryo transfers, mainly because only the effect of embryo quality in pregnancy rates would be evaluated, and the effects of Dual Trigger or hCG alone would not be present on the endometrial receptivity. In the papers evaluated, there was a variation between the dosages of hCG. In the groups of poor responder patients, from 5,000 IU to 10,000UI, regardless of the dose, the results were always positive concerning the use of Dual Trigger (Zilberberg *et al*., 2015; Griffin *et al*., 2012). It is worth mentioning that in all the studies, it was necessary to provide an intensive support during the luteal phase, and some of the studies did use high doses of progesterone alone, or estrogen and progesterone in the luteal phase (Decleer *et al*., 2014). This undermines the certification, in practice, of the theory that the Dual Trigger improves the endometrial receptivity, perhaps, this improvement in implantation rates has been produced by high doses of progesterone.

The Dual Trigger, to induce oocyte maturation has the advantage of acting more physiologically as it induces an FSH surge. The importance of FSH present in the oocyte maturation process has been proved in a large number of studies that the GnRH agonist increases the number of mature oocytes (Zeleznik *et al*., 1974; Richards *et al*., 1976). Lamb *et al.* (2011) carried out a study using an FSH bolus concurrently with the use of hCG as a trigger, and found an increased number of mature oocytes and fertilization rate after the use of FSH (19). Our study, as well as the majority of the studies investigated in this paper, was limited by its small sample size, requiring, however, a randomized prospective study with a larger number of patients for more solid conclusions about the use of Dual Trigger.

In conclusion, according to this retrospective study, the Dual Trigger used for induction of final oocyte maturation in normal responders, poor responders, and patients with immature oocytes, significantly improves the number of collected oocytes, mature oocytes, and fertilized embryos, as well as improves the beta hCG positive rate, and the implantation and pregnancy rates. The results presented in this article reinforce the evidence of improved outcomes of Human Reproduction treatments using this method.
